# Supplementary material for: Parallel sequencing of extrachromosomal circular DNAs and transcriptomes in single cancer cells
Source: Nat Genet. 2023 May 4;55(5):880–90. doi: 10.1038/s41588-023-01386-y (PMC10181933; doi:10.1038/s41588-023-01386-y)
Supplement: Supplementary file 1 — Supplementary Figs. 1–18, Table 7 and Note 1. [file 41588_2023_1386_MOESM1_ESM.pdf]

nature genetics

Technical Report

https://doi.org/10.1038/s41588-023-01386-y

Parallel sequencing of extrachromosomal circular DNAs and transcriptomes in single cancer cells

In the format provided by the authors and unedited

Supplementary information for Chamorro et al. "Parallel sequencing of extrachromosomal circular DNAs and transcriptomes in single cancer cells"

Rocío Chamorro González[1,2], Thomas Conrad[18], Maja C. Stöber[3,4,17], Robin Xu[1,2], Madalina Giurgiu[1,2,5], Elias Rodriguez-Fos[1,2], Katharina Kasack[6], Lotte Brückner[2,19], Eric van Leen[1,2], Konstantin Helmsauer[1,2], Heathcliff Dorado Garcia[1,2], Maria E. Stefanova[7,8], King L. Hung[9], Yi Bei[1,2], Karin Schmelz[1], Marco Lodrini[1], Stefan Mundlos[7,8,10], Howard Y. Chang[9,11], Hedwig E. Deubzer[1,2,12,13], Sascha Sauer[3], Angelika Eggert[1,12,13], Johannes H. Schulte[1,12,13], Roland F. Schwarz[14,15,3], Kerstin Haase[1,2,12], Richard P. Koche[16*] and Anton G. Henssen[1,2,12,19*]

[1]Department of Pediatric Oncology and Hematology, Charité – Universitätsmedizin Berlin, corporate member of Freie Universität Berlin, Humboldt-Universität zu Berlin, Berlin, Germany.

[2]Experimental and Clinical Research Center (ECRC) of the MDC and Charité Berlin, Berlin, Germany.

[3]Berlin Institute for Medical Systems Biology (BIMSB), Max Delbrück Center for Molecular Medicine in the Helmholtz Association, Berlin, Germany.

[4]Charité—Universitätsmedizin Berlin, Berlin, Germany.

[5]Freie Universität Berlin, Berlin, Germany.

[6]Fraunhofer Institute for Cell Therapy and Immunology, Branch Bioanalytics and Bioprocesses IZI-BB, Potsdam, Germany.

[7]RG Development and Disease, Max Planck Institute for Molecular Genetics, Berlin, Germany

[8]Institute for Medical Genetics, Charité – Universitätsmedizin Berlin, Germany

[9]Center for Personal Dynamic Regulomes, Stanford University School of Medicine, Stanford, CA, USA

[10]Berlin-Brandenburg Center for Regenerative Therapies (BCRT), Charité – Universitätsmedizin Berlin, Germany

[11]Howard Hughes Medical Institute, Stanford University School of Medicine, Stanford, CA, USA

[12]German Cancer Consortium (DKTK), partner site Berlin, and German Cancer Research Center (DKFZ), Heidelberg, Germany.

[13]Berlin Institute of Health, 10178 Berlin, Germany.

[14]Institute for Computational Cancer Biology (ICCB), Center for Integrated Oncology (CIO), Cancer Research Center Cologne Essen (CCCE), Faculty of Medicine and University Hospital Cologne, University of Cologne, Germany.

[15]Berlin Institute for the Foundations of Learning and Data (BIFOLD), Berlin, Germany.

[16]Center for Epigenetics Research, Memorial Sloan Kettering Cancer Center, New York, New York, USA.

[17]Humboldt-Universität zu Berlin, Faculty of Life Science, 10099 Berlin, Germany.

[18]Genomics Technology Platform, Max Delbrück Center for Molecular Medicine in the Helmholtz Association (MDC), Berlin, Germany

[19]Max-Delbrück-Centrum für Molekulare Medizin, Berlin, Germany.

*These authors jointly supervised this work.

Correspondence should be addressed to A.G.H. (henssenlab@gmail.com).

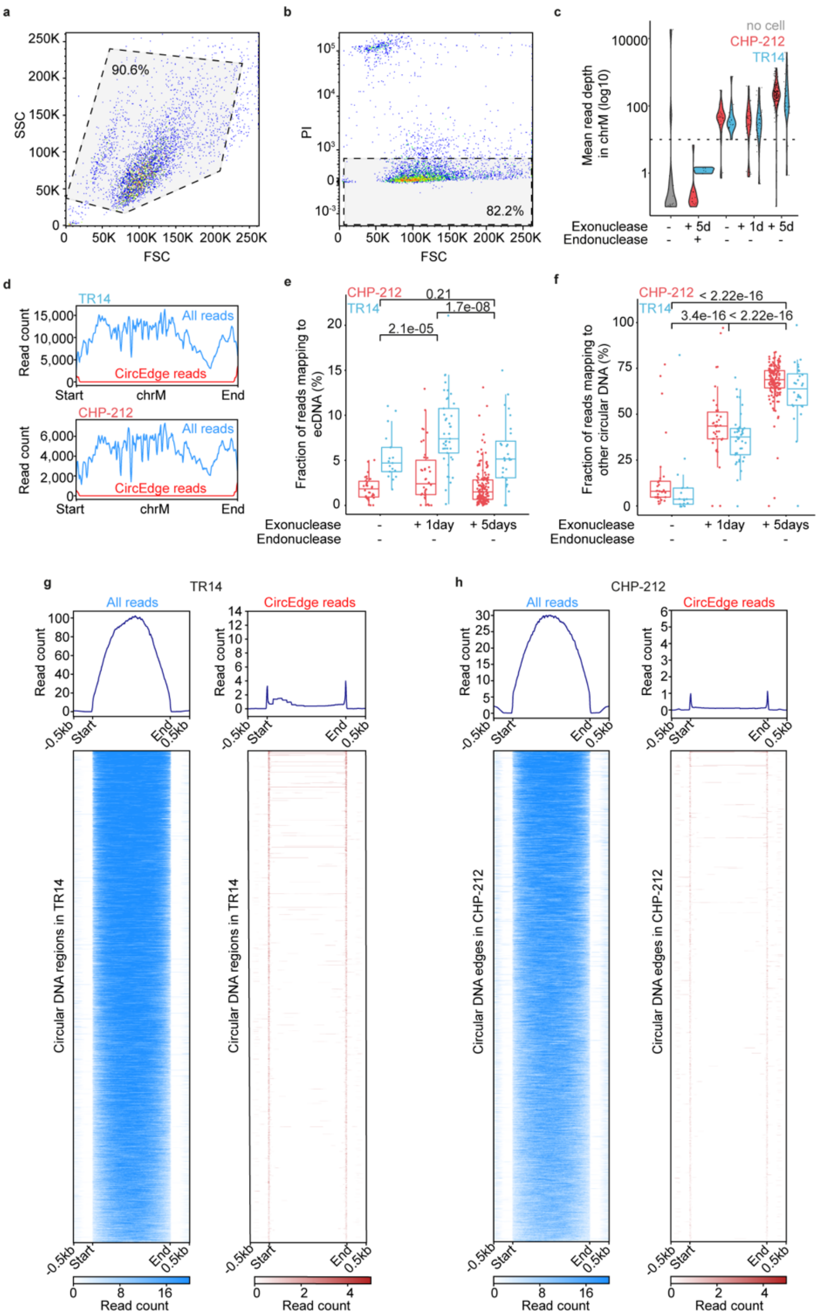

Supplementary Figure 1. Quality control of single cell Circle-seq data (scCircle-seq). **a-b,** Results from Fluorescence-activated cell sorting (FACS) of CHP-212 cells. Forward scatter (FSC) vs. side scatter (SSC) plot (a) with gate (dotted line) to separate events from debris. Forward scatter (FSC) vs. propidium iodide (PI) plot (b) with gate (dotted line) to separate live cells from dead cells. Cell percentages are shown. **c,** Violin plot showing per base-pair mean read depth in mitochondrial DNA (chrM) in TR14 cells (blue; $n = 17$ non digested cells, $n = 41$ one-day exonuclease digested cells, $n = 28$ five-days exonuclease digested cells, $n = 6$ endonuclease and exonuclease digested cells) and CHP-212 cells (red; $n = 30$ non digested cells, $n = 38$ one-day exonuclease digested cells, $n = 154$ five-days exonuclease digested cells, $n = 12$ endonuclease and exonuclease digested cells) and empty wells (no cell, grey; $n = 7$). For QC filtering of scCircle-seq data, we used a threshold of minimum 10 per base read depth (dotted line). **d**, Total read (blue line) and circle-edge split read count density (red line) over mtDNA (chrM) in merged TR14 cells (top) and merged CHP-212 cells (bottom). **e**, Fraction of sequencing reads

mapping to ecDNA regions in each experimental condition in CHP-212 and TR14 cells. **f,** Fraction of sequencing reads mapping to other circular DNA regions (excluding ecDNA regions and mtDNA) in each experimental condition in CHP-212 and TR14 cells. In figs. e and f, sample size is identical across conditions: no digestion (*n* = 16 TR14 cells, *n* = 28 CHP-212 cells), one-day exonuclease digestion (*n* = 37 TR14 cells, *n* = 31 CHP-212 cells), five-days exonuclease digestion (*n* = 25 TR14 cells, *n* = 150 CHP-212 cells) and endonuclease digestion with PmeI prior to five-days exonuclease digestion (*n* = 6 TR14 cells, *n* = 12 CHP-212 cells). **g,h,** Total read and circle-edge split read count density over individual circular DNA regions identified by scEC&T-seq in merged TR14 cells (e) and merged CHP-212 cells (f). Top left and right: cumulative plots of total read count (left) and circle-edge split read count (right) density over all circular DNA regions; bottom left and right: heat map of total read count (left, blue) and circle-edge split read count (right, red) in all identified circular DNA regions. All statistical analyses correspond to two-sided Welch's *t*-test. In all boxplots, boxes represent 25th and 75th percentile with center bar as median value and whiskers represent furthest outlier ≤1.5× the interquartile range from the box.

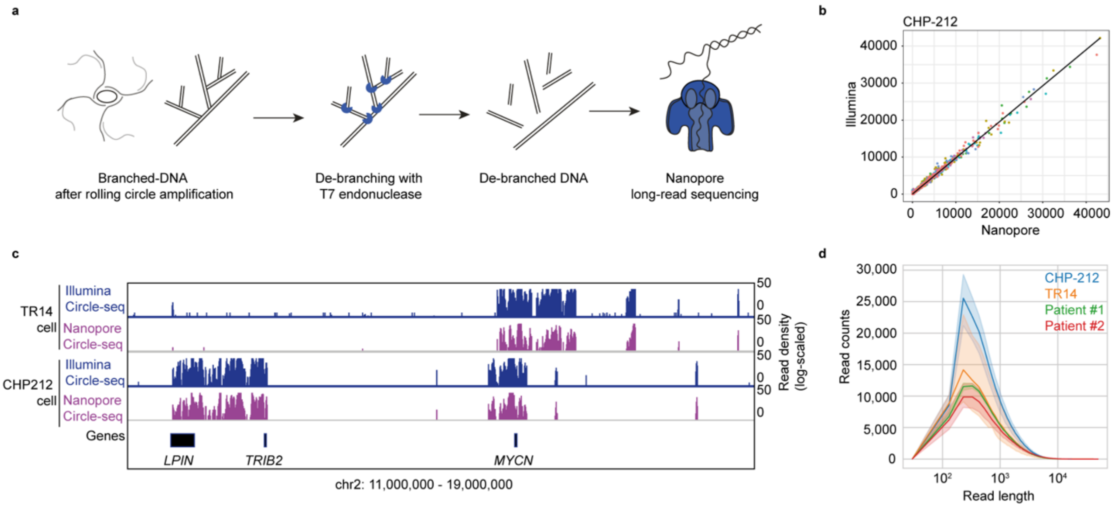

Supplementary Figure 2. Nanopore-based detection of extrachromosomal circular DNAs in single cells. a, Schematic of T7 endonuclease de-branching of rolling-circle amplified DNA prior to nanopore Circle-seq. **b**, Correlation of normalized read counts from Illumina and Nanopore scCircle-seq data from a subset of CHP-212 cells (log-scaled, two-sided Pearson correlation: $R = 0.95$, $P < 2.2e\text{-}16$). Each color represents a different cell, and each point is a putative circle. **c**, Genome tracks comparing log-scaled read coverage across the *MYCN* ecDNA amplicon regions in Illumina (blue) vs Nanopore (pink) Circle-seq data in two exemplary cells (CHP-212 and TR14). **d,** Read length distribution of Nanopore sCircle-seq data. Individual lines represent the average across single cells grouped by sample (n = 6 CHP-212 cells (blue), n = 3 TR-14 cells (orange), n = 4 patient #1 nuclei (green), n = 5 patient #2 nuclei (red)), whereas the shade stands for 95% confidence interval.

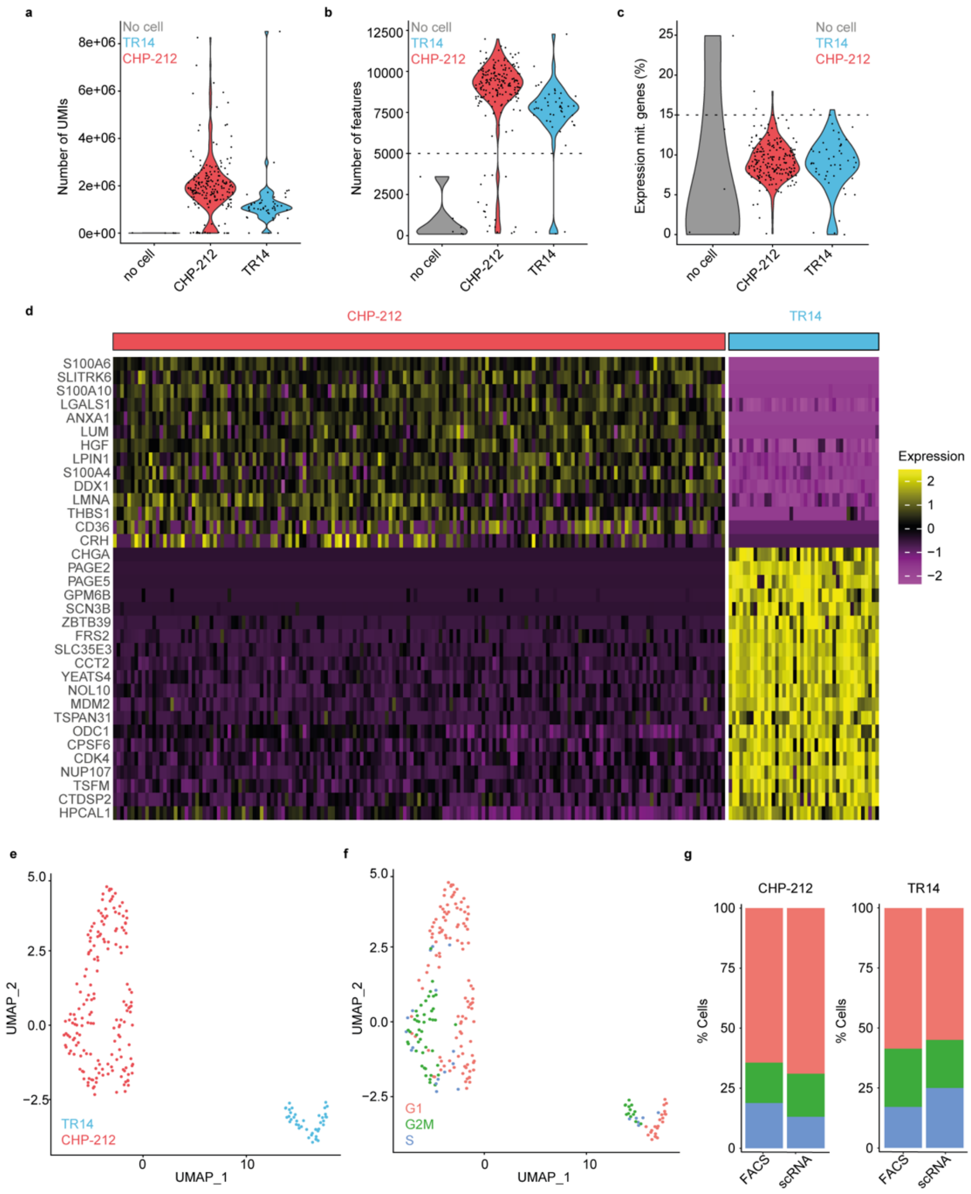

Supplementary Figure 3. **Quality control of scRNA-seq data. a,** Violin plot of number of unique molecular identifiers (UMIs) in CHP-212 (red; *n* = 171) and TR14 (blue; *n* = 42) cells and in negative controls (grey; *n* = 5). **b,** Violin plot of number of features (genes) identified in CHP-212 (red; *n* = 171; mean +/- sd = 9,328 +- 1,006) and TR14 (blue; *n* = 42; mean +/- sd = 7,961 +- 1,124) single cells and in negative controls (grey; *n* = 5). **c,** Violin plot of fraction of expression (%) of mitochondrial genes in CHP-212 (red; *n* = 171) and TR14 (blue; *n* = 42) single-cells and in negative controls (grey; *n* = 5). **d,** Heatmap showing the top 20 differentially expressed genes between CHP-212 and TR14 cells (*n* = 171 CHP-212 cells in red, *n* = 42 TR14 cells in blue). **e,** UMAP visualization showing clusters of transcriptionally similar cells colored by cell line identity (*n* = 171 CHP-212 cells in red, *n* = 42 TR14 cells in blue). **f,** UMAP visualization illustrating cells from **e** colored by predicted cell cycle phase (G1 in red, G2M in green, S in blue). **g,** Bar plots comparing the relative distribution of CHP-212 and TR14 cells (%) across cell cycle phases measured by FACS-based cell cycle analysis (PI) or inferred from scRNA-seq data (G1 in red, G2M in green, S in blue).

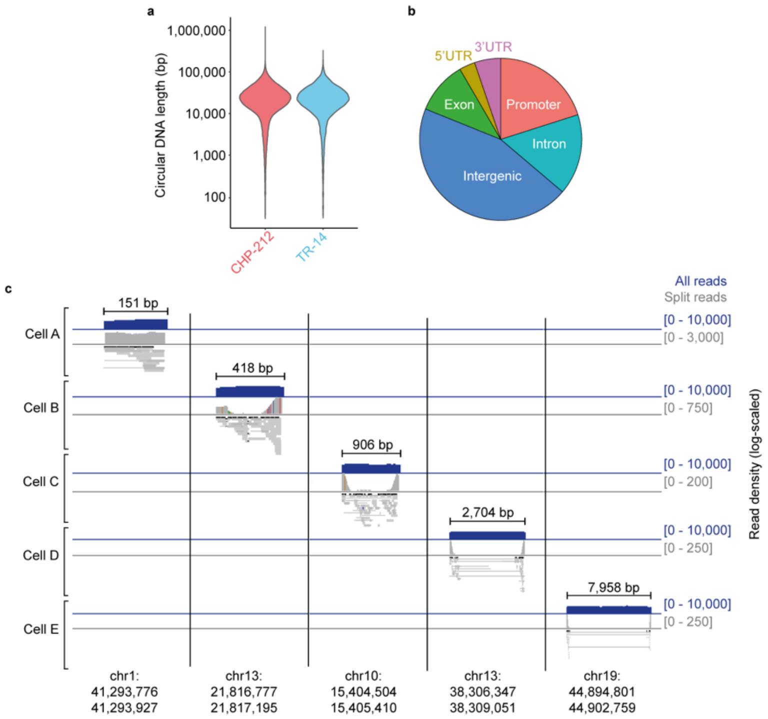

Supplementary Figure 4. The majority of small extrachromosomal circular DNAs are not recurrently identified in single cells a, Size distribution of extrachromosomal circular DNAs identified using scEC&T-seq in CHP-212 and TR14 single cells (log-scaled, *n* = 150 CHP-212 cells, *n* = 25 TR14 cells). **b,** Fraction of genomic regions affected by extrachromosomal DNA circularization in TR14 (*n* = 25 cells) and CHP-212 (*n* = 150 cells) (promoter = 20.11% (red), intron = 16.10% (light blue), intergenic = 44.88% (dark blue), exon = 10.51% (green), 5'UTR = 3.16% (brown), 3'UTR = 5.24% (pink)). **c,** Exemplary genome tracks of non-recurrent extrachromosomal DNA circularization in 5 different CHP-212 single cells. Log-scaled total read density is shown in blue and log-scaled circle edge read density is shown in grey.

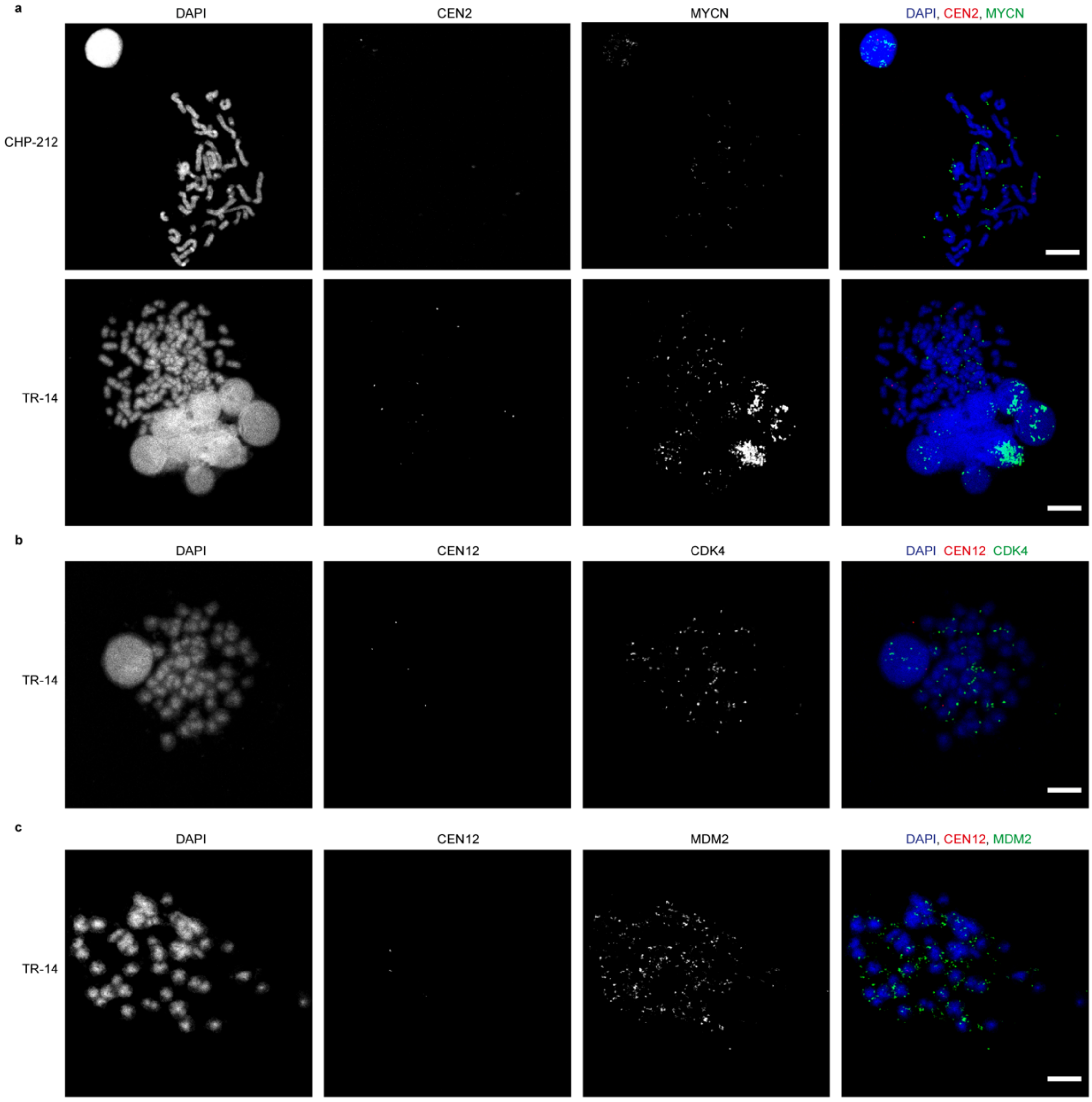

Supplementary Figure 5. Fluorescence in situ hybridization in neuroblastoma cell lines. a, DNA - fluorescence in situ hybridization (FISH) of CHP-212 (top row) and TR14 (bottom row) metaphase spreads with *MYCN* probe (green) and control chromosomal probe for chromosome 2 (CEN2; red). Nuclei and chromosomes are stained with DAPI (blue). Channels left to right: DAPI, CEN2, *MYCN* and merged. **b,** FISH of TR14 metaphase spreads with *CDK4* probe (green) and control chromosomal probe for chromosome 12 (CEN12; red). Nuclei and chromosomes are stained with DAPI (blue). Channels left to right: DAPI, CEN12, *CDK4* and merged. **c,** FISH of TR14 metaphase spreads with *MDM2* probe (green) and control chromosomal probe for chromosome 12 (CEN12; red). Nuclei and chromosomes are stained with DAPI (blue). Channels left to right: DAPI, CEN12, *MDM2* and merged. In all cases, the scale bar is 10 µm. FISH experiments were done once per cell line.

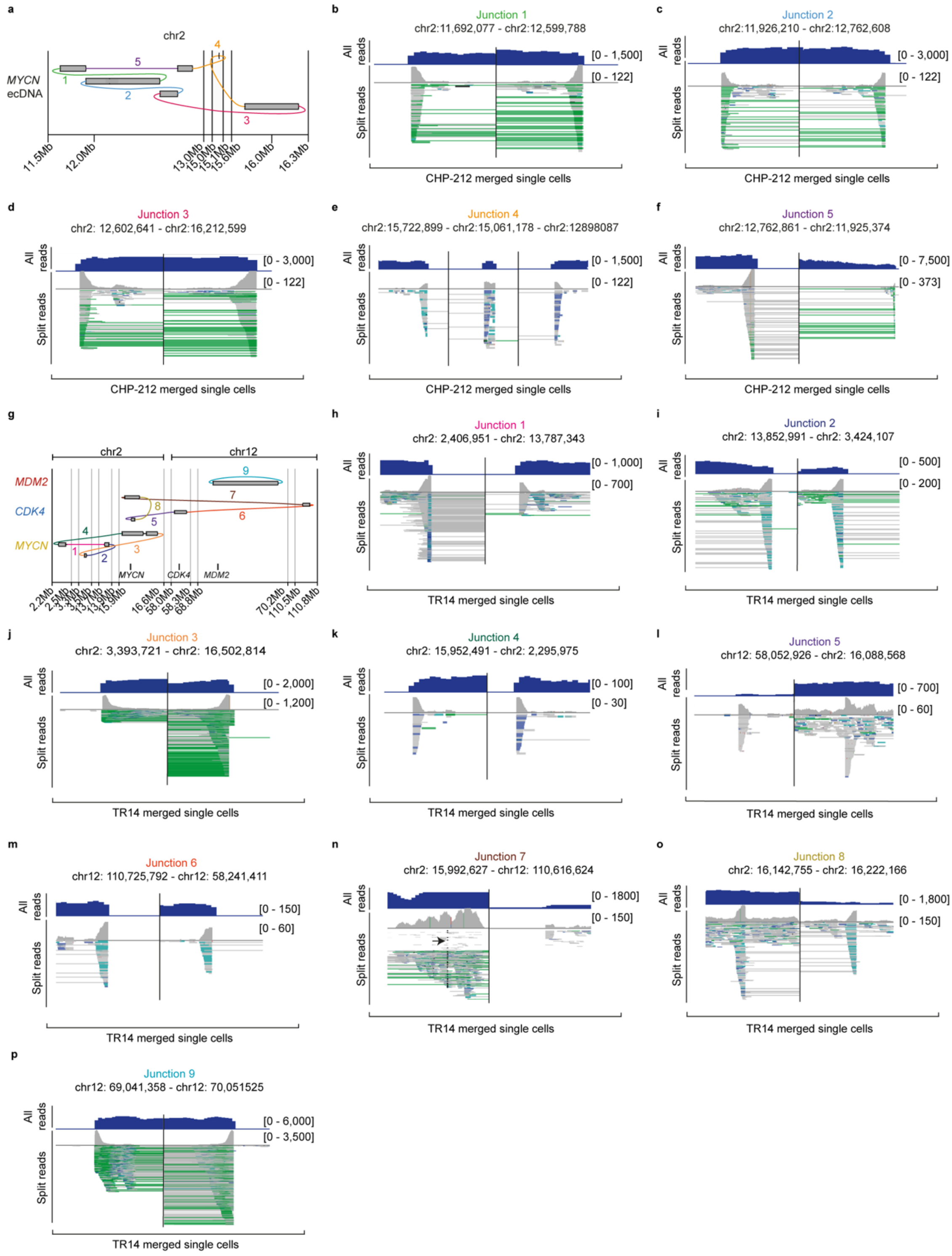

Supplementary Figure 6. Detection of ecDNA junction-supporting split reads in scEC&T-seq data. a, Long and short-read based ecDNA amplicon reconstruction from whole-genome bulk sequencing data in CHP-212. (**b-f**), Total and split read density at predicted junctions in merged CHP-212 scEC&T-seq data. **g,** Long and short-read based ecDNA amplicon reconstructions from whole-genome bulk sequencing data in TR14. (**h-p**), Total and split read density at predicted junctions in merged CHP-212 scEC&T-seq data.

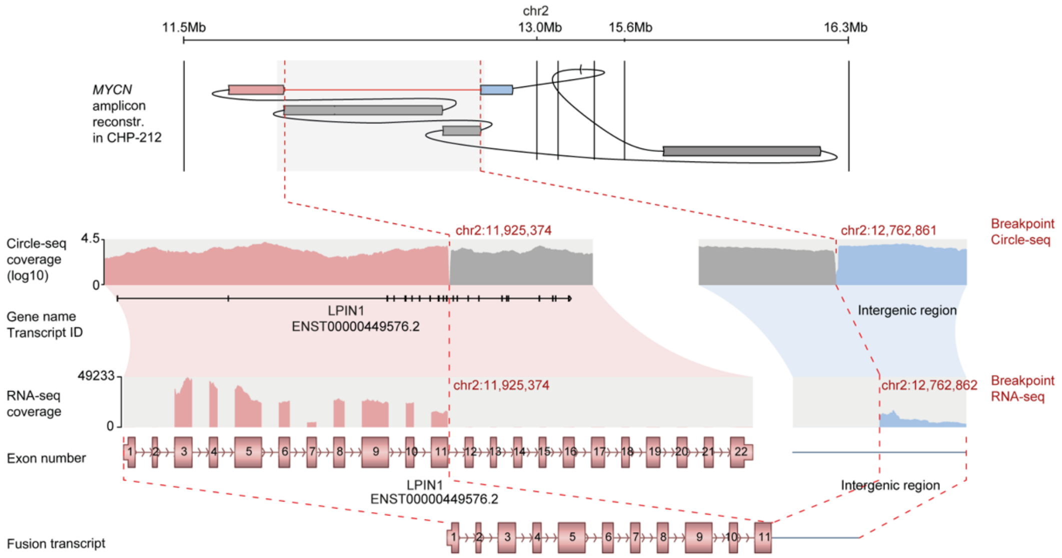

Supplementary Figure 7. Detection of fusion transcript from scRNA-seq data. Example of a fusion transcript resulting from the rearrangement of chromosomal segments in the *MYCN* ecDNA in CHP-212 cells. Top to bottom: amplicon reconstruction from WGS data, Circle-seq read coverage over the breakpoint region in merged CHP-212 single cells (log-scaled), transcript annotations, merged scRNA-seq read coverage over the fused transcripts, native transcripts representations, fusion transcript representation.

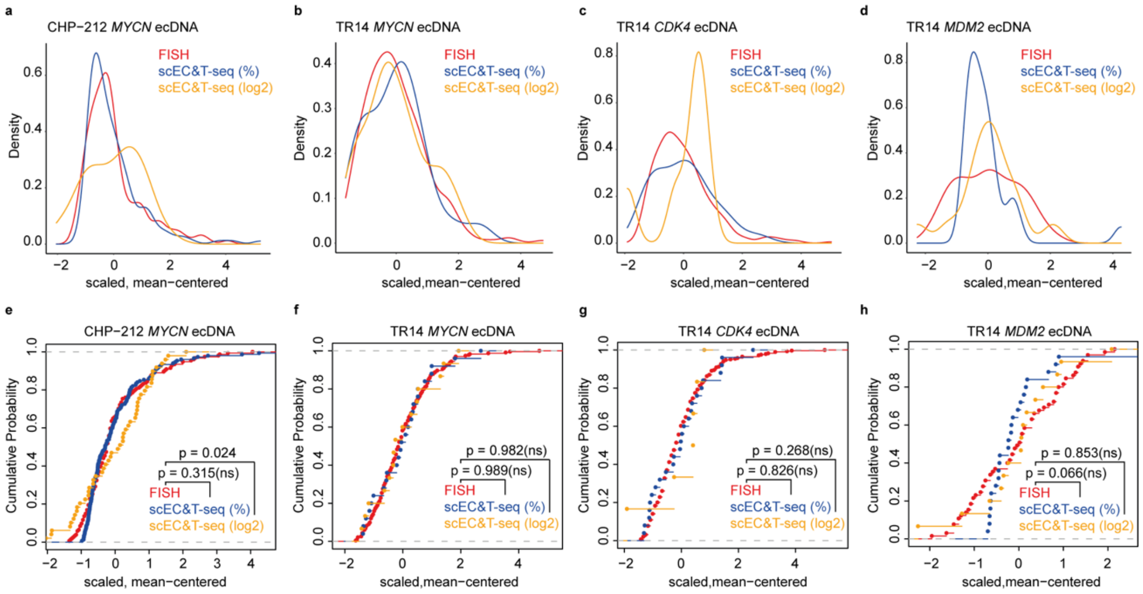

Supplementary Figure 8: Relative ecDNA copy number measured using scEC&T-seq resembles FISH-based copy number estimates. a-d, Density plots displaying the scaled, mean-centered ecDNA relative copy number distributions in CHP-212 (a, *MYCN* ecDNA) and TR14 (b, *MYCN* ecDNA; c, *CDK4* ecDNA; d, *MDM2* ecDNA), as measured by *MYCN* DNA interphase FISH (red, *n* =154 (a), *n* = 232 (b), *n* = 284 (c), *n* = 65(d)), log2 *MYCN* coverage in scEC&T-seq (yellow, *n* = 49 (a), *n* = 15 (b), *n* = 6 (c), *n* = 15(d)) and fraction of ecDNA-specific reads in scEC&T-seq (blue, *n* = 150 (a), *n* = 25 (b-d)). **e-h,** Cumulative probability of scaled, mean-centered ecDNA relative copy number in CHP-212 (a, *MYCN* ecDNA) and TR14 (b, *MYCN* ecDNA; c, *CDK4* ecDNA; d, *MDM2* ecDNA), as measured by *MYCN* DNA interphase FISH (red, *n* = 154 (a), *n* = 232 (b), *n* = 284 (c), *n* = 65(d)), log2 *MYCN* coverage in scEC&T-seq (yellow, *n* = 49 (a), *n* = 15 (b), *n* = 6 (c), *n* = 15(d)) and fraction of ecDNA-specific reads in scEC&T-seq (blue, *n* = 150 (a), *n* = 25 (b-d)). P-values were calculated by Kolmogorov-Smirnov test and are shown. FISH experiments were done once per cell line.

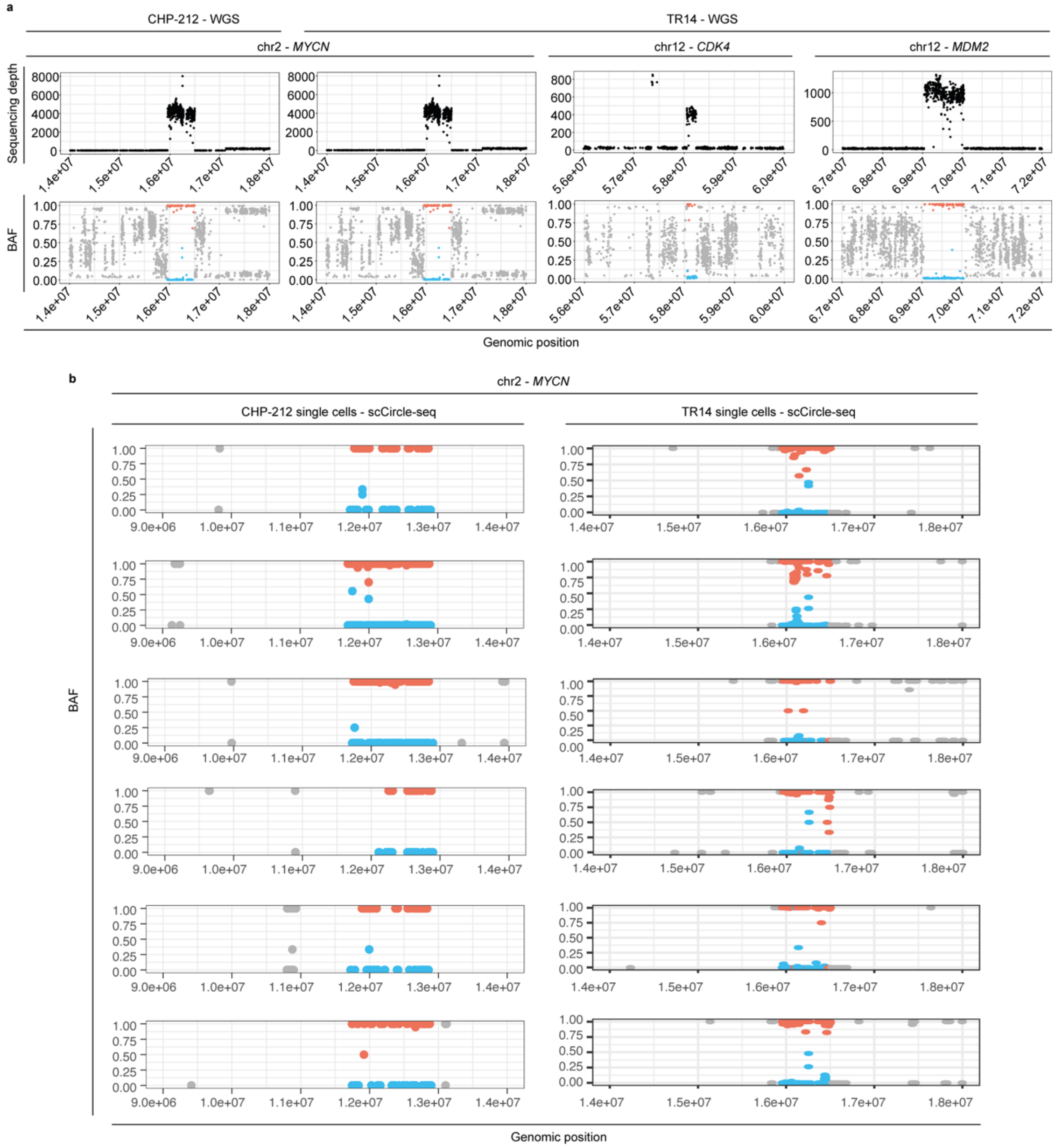

Supplementary Figure 9. Phasing of SNPs in ecDNA loci in scEC&T-seq data indicates ecDNAs are of mono-allelic origin. a, Reference phasing of *MYCN, CDK4* and *MDM2* ecDNA loci in bulk WGS data. Shown is the raw sequencing coverage (top) and the B-allele frequency (BAF; bottom) of known SNPs based on the 1000 genomes annotation. SNPs in regions of high-level amplifications can be very clearly assigned to the gained or non-gained allele based on BAF. **b,** Genotyped *MYCN* ecDNA locus in scCircle-seq CHP-212 and TR14 sequencing data (6 exemplary cells in each case are shown). Shown is the B-allele frequency (BAF) of known SNPs based on the 1000 genomes annotation. SNPs that have been reference phased based on bulk sequencing data are colored the same as in (a).

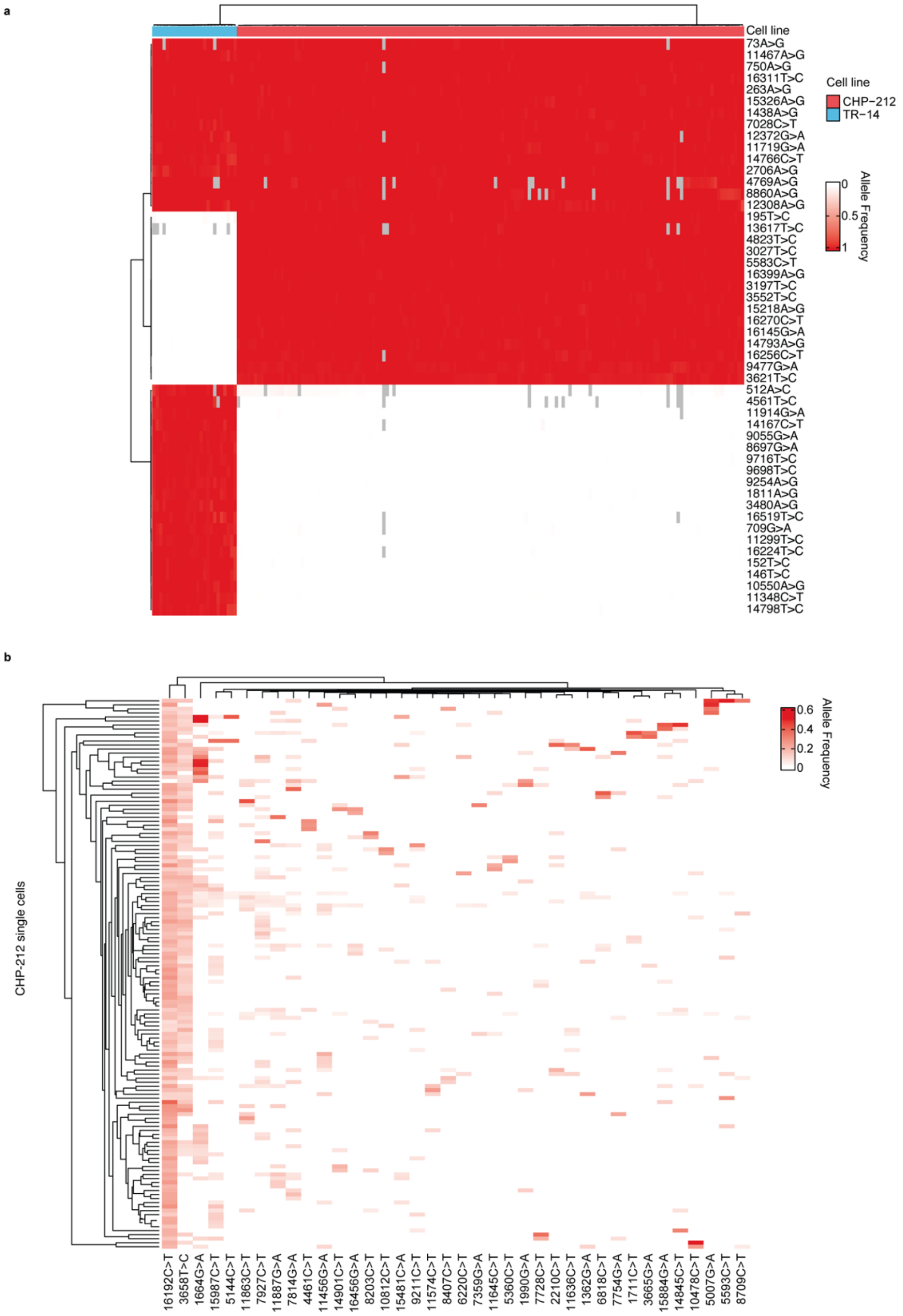

Supplementary Figure 10. scEC&T-seq enables identification of homoplasmic and heteroplasmic variants (SNVs) in mitochrondrial DNA. a, Homoplasmic mitochondrial single nucleotide variants (SNV) detected in CHP-212 and TR14 single cells (n = 150 CHP-212 cells, n = 25 TR14 cells). Unsupervised hierarchical clustering allows for clear separation of both cell lines based on their haplogroup variants, suggesting usage for population scale phylogeny studies. Sites with read depth ≤ 10 are shown in grey. **b,** Heteroplasmic variants detected in CHP-212 single cells (n = 150 CHP-212 cells). Unsupervised hierarchical clustering (y-axis) suggests usage for lineage tracing exploration and applications. Sites with read depth ≤ 10 are shown in grey.

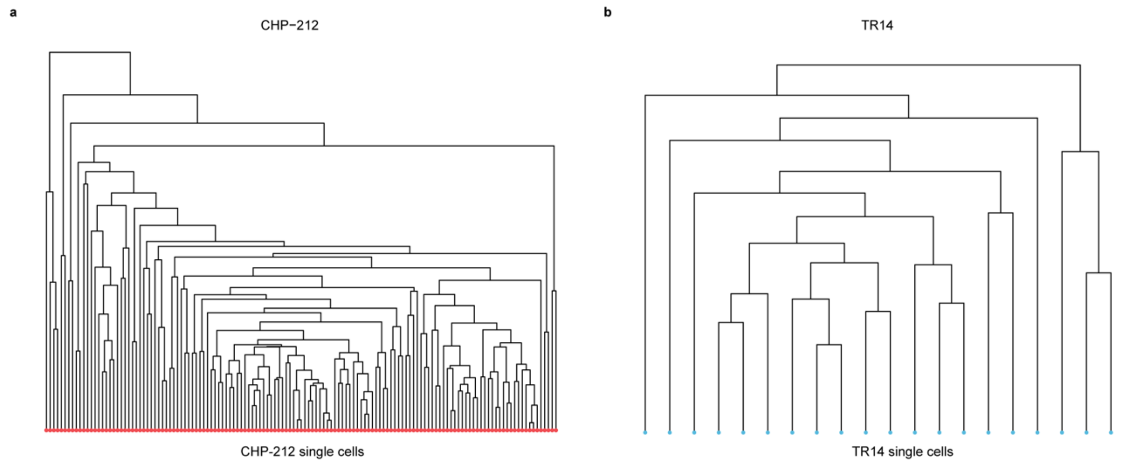

Supplementary Figure 11. **Mitochondrial heteroplasmic SNVs can be used to infer phylogeny. a,b,** Phylogenetic trees inferred from heteroplasmic single-nucleotide variants identified in mitochondrial DNA in CHP-212 (a; n = 148) and TR14 (b; n = 20) single cells.

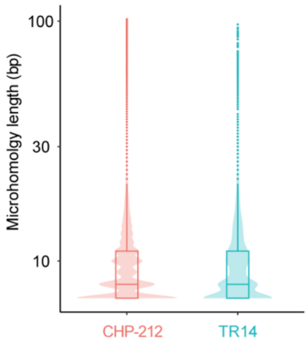

Supplementary Figure 12. **Microhomology detection at circular DNA breakpoints. a,** Length distribution of microhomologies in CHP-212 and TR14 single cells (*n* = 150 CHP-212 cells and *n* = 25 TR14 cells). All boxplot's box represents 25th and 75th percentile with center bar as median value and whiskers represent furthest outlier ≤1.5× the interquartile range from the box.

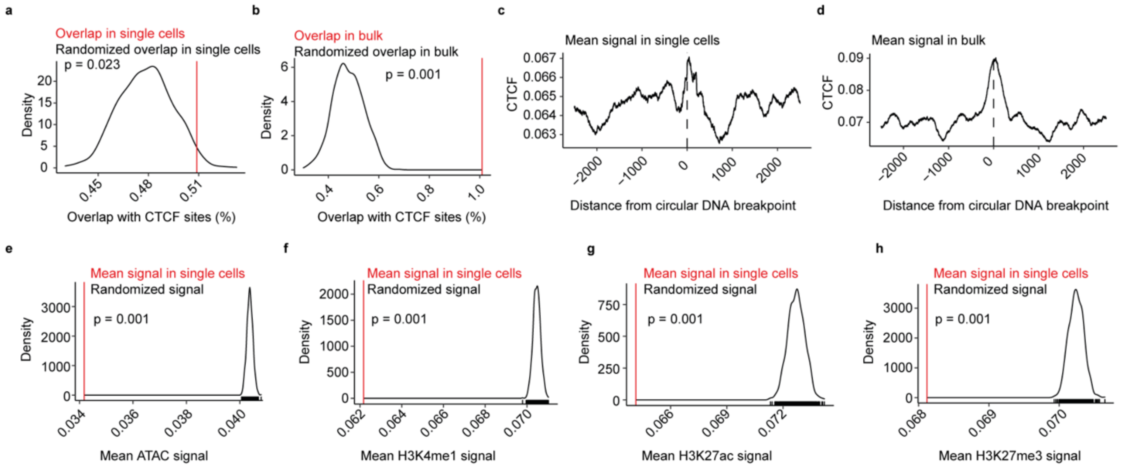

Supplementary Fig. 13. **Chromatin marks and chromatin accessibility in extrachromosomal circular DNAs in single cells. a, b,** Fraction of circular DNA edge regions overlapping with CTCF ChIP-seq peaks in CHP-212 single cells (a) and in bulk CHP-212 Circle-seq (b). Overlap shown in red and randomized overlap in dark**. c, d,** Mean CTCF signal around the edges (dashed line) of circular DNA regions detected in all CHP-212 single cells (c) and in bulk CHP-212 Circle-seq (d). **e-h,** Mean ChIP-seq or ATAC-seq signal across all detected circular DNA regions in all CHP-212 single cells (red) and randomized signal (black): ATAC-seq (e, *P* = 1e-06); H3K4me1 ChIP-seq (f, *P* = 0.001); H3K27ac ChIP-seq (g, *P* = 1e-06); H3K27me3 ChIP-seq (h, *P* = 0.494). Empirical one-sided p-values were used from randomization analyses.

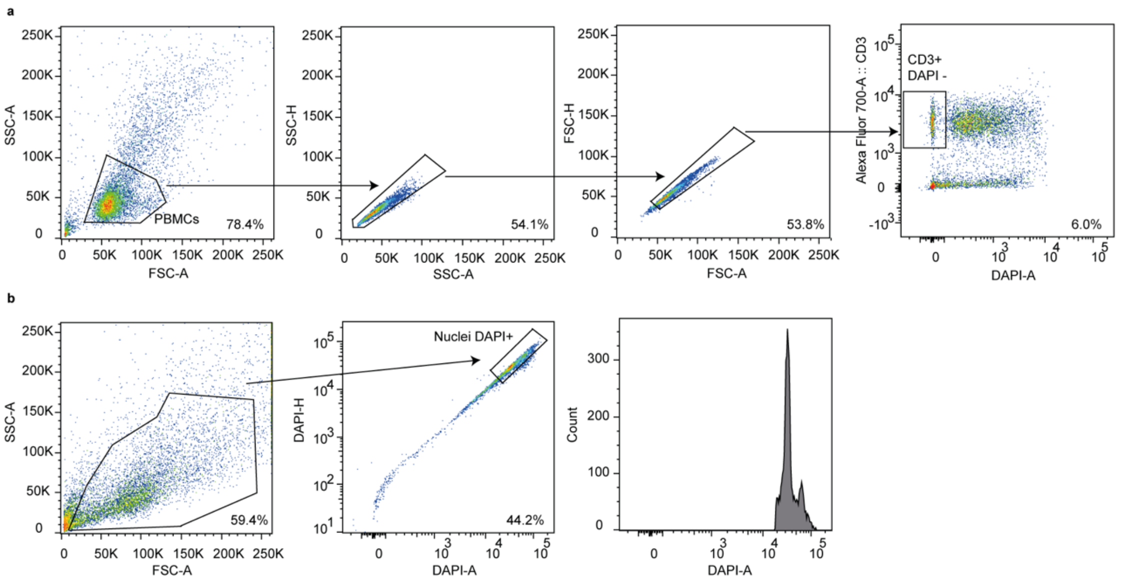

Supplementary Fig. 14: Representative FACS gating strategy for PBMCs and nuclei. a, Representative gating strategy of CD3+ DAPI- live T-cells from PBMCs population derived from patient's blood in patient #3. **b,** Representative gating strategy for nuclei isolated from primary tumor samples. In both cases, Forward scatter (FSC) and side scatter (SSC) were used to separate events from debris. DAPI, in PBMCs, was used to stain and filter out dead cells. DAPI+ nuclei were sorted. Gating strategy and cell percentages are shown in each case.

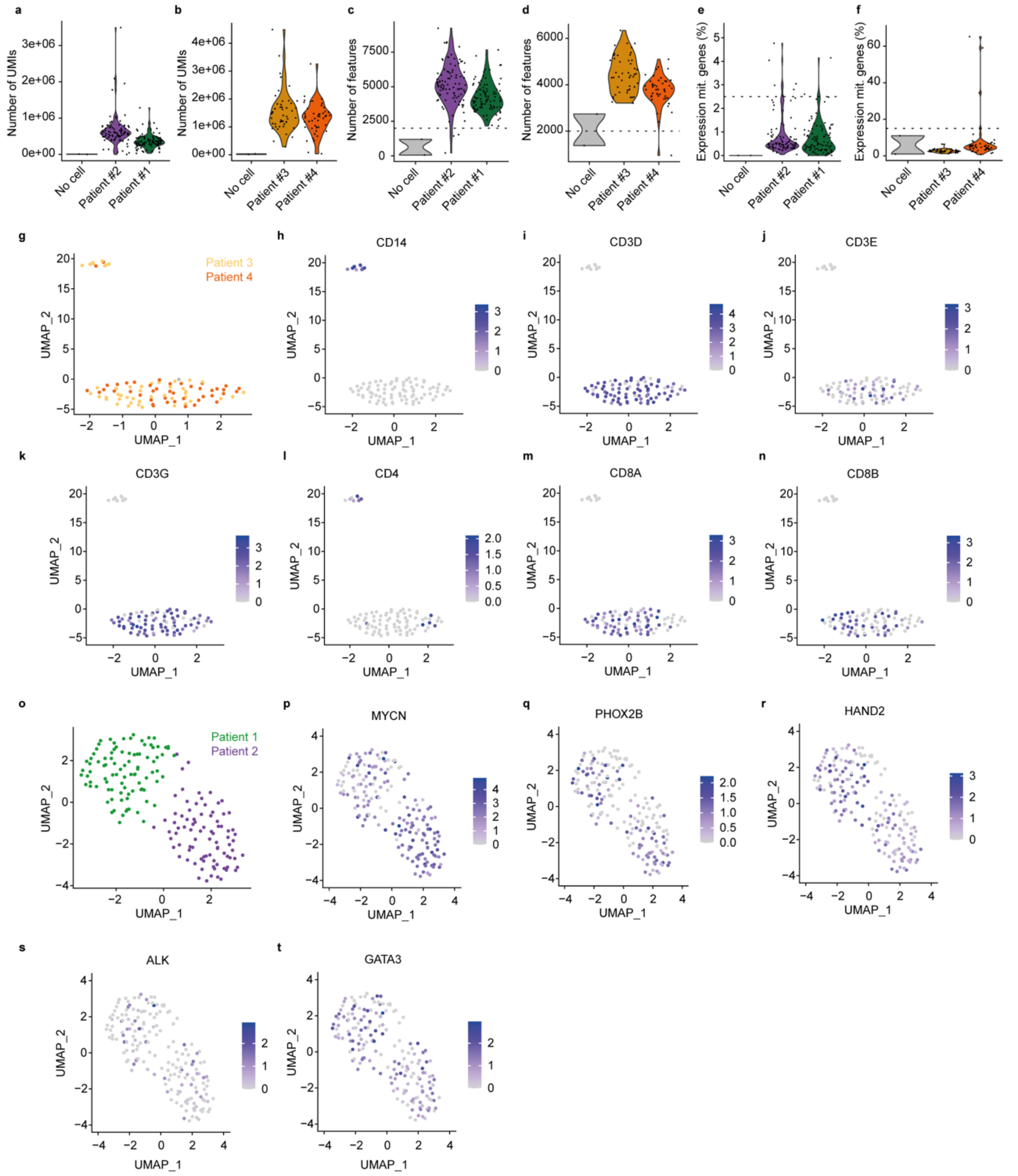

Supplementary Figure 15. Quality control of tumor and T-cell scRNA-seq data. a,b, Violin plot of number of unique molecular identifiers (UMIs) in primary tumor nuclei (a, n = 93 nuclei patient #1, green; n = 86 nuclei patient #2, purple) and single T-cells (b, n = 38 patient #3, yellow; n = 41 patient #4, orange). **c,d,** Violin plot of number of features (genes) identified in primary tumor nuclei (c, n = 93 nuclei patient #1, green; n = 86 nuclei patient #2, purple) and single T-cells (b, n = 38 patient #3, yellow; n = 41 patient #4, orange). **e,f,** Violin plot of fraction of expression (%) of mitochondrial genes in primary tumor nuclei (e, n = 93 nuclei patient #1, green; n = 86 nuclei patient #2, purple) and single T-cells (b, n = 38 patient #3, yellow; n = 41 patient #4, orange). **g,** UMAP visualization showing clusters of transcriptionally similar cells colored by patient identity (n = 38 patient #3 shown in yellow, n = 41 patient #4 shown in orange). **h-n,** UMAP visualization showing relative expression of marker genes: *CD14* (h), *CD3D* (i), *CD3E* (j), *CD3G* (k), *CD4* (l), *CD8A* (m), *CD8B* (n). **o,** UMAP visualization showing clusters of transcriptionally similar nuclei colored by patient identity (n = 93 nuclei patient #1

shown in green, n = 86 nuclei patient #2 shown in purple). **p-t,** UMAP visualization showing relative expression of neuroblastoma marker genes: *MYCN* (p), *PHOX2B* (q), *HAND2* (r), *CD3G* (k), *ALK* (s), *GATA3* (t).

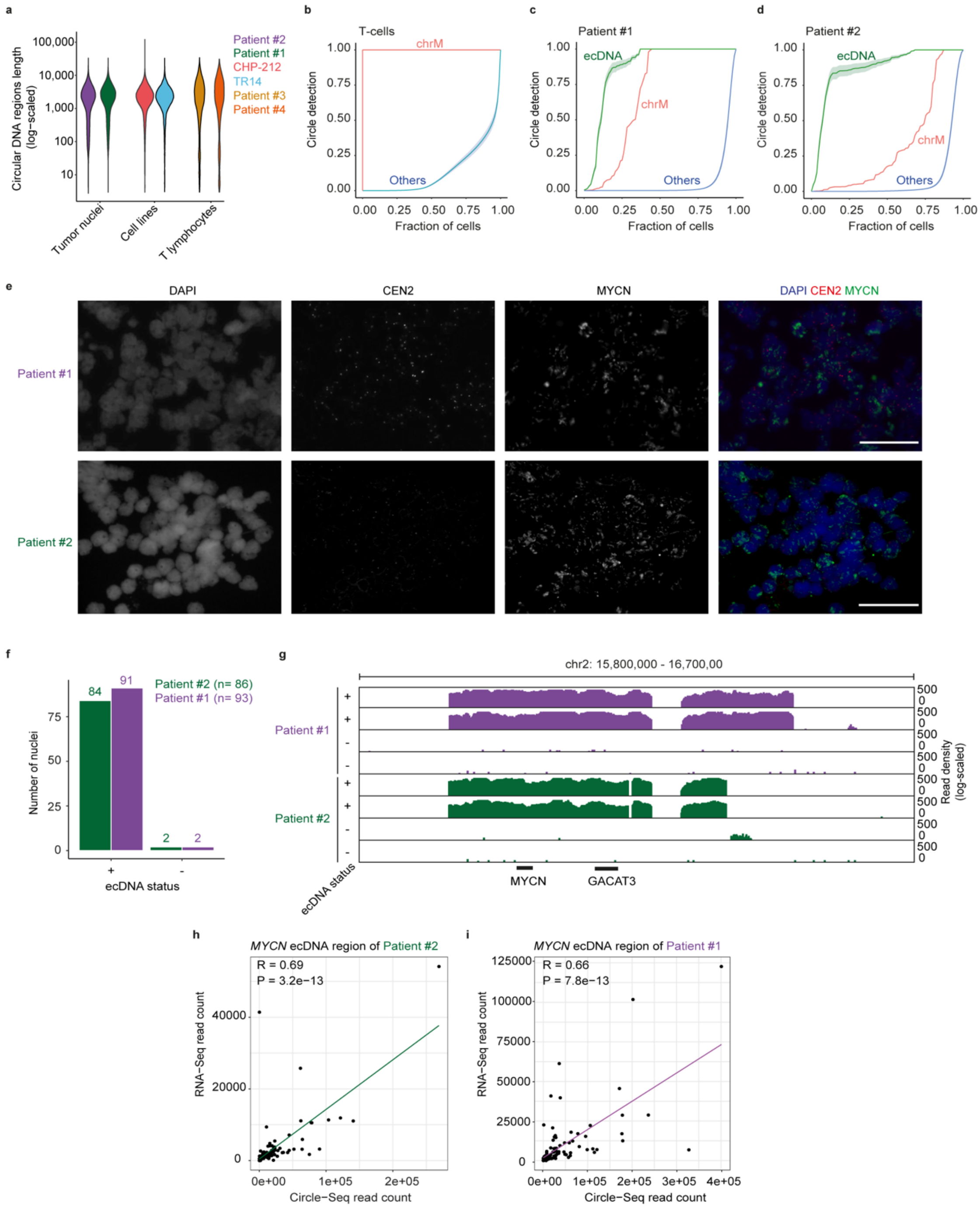

Supplementary Figure 16. Intercellular differences in ecDNA content in primary neuroblastoma tumors drive gene expression differences. a, Log-scaled size distribution of extrachromosomal circular DNAs identified using scEC&T-seq in primary tumor nuclei (n = 93 nuclei patient #1 shown in green; n = 86 nuclei patient #2 shown in purple), neuroblastoma cell lines (n = 150 CHP-212 in red, n = 25 TR14 in blue) and single T-cells (n = 38 patient #3 shown in yellow; n = 41 patient #4 shown in orange).**b-d,** Recurrence analysis in single T-cells (b, n = 79 T-cells), primary tumor nuclei from patient #1(c, n = 93 nuclei) and pantient #2(d, n = 86 nuclei) displayed as fraction of cells containing a detected circular DNA from each circular DNA type. ecDNA was defined as extrachromosomal circular DNAs overlapping with copy number amplified regions identified in bulk sequencing (green); chrM (red); "Others" are defined as all other

extrachromosomal circular DNAs (blue). Data are presented as mean values +/- SEM. **e,** Interphase FISH of patient #1 (top row) and patient #2 (bottom row) with *MYCN* probe (green) and control chromosomal probe for chromosome 2 (CEN2; red). Nuclei are stained with DAPI (blue). Channels left to right: DAPI, CEN2, *MYCN* and merged. Scale bar indicates 50 µm. **f,** Bar plot of number of nuclei based on ecDNA status (n = 93 nuclei patient #1 shown in green; n = 86 nuclei patient #2 shown in purple). **g,** Genome tracks with read densities (log-scaled) over reconstructed *MYCN* ecDNA region in 8 exemplary patient #1(green) and #2(purple) nuclei showing + and - ecDNAs status. **h,i,** Pairwise comparison between ecDNA and mRNA read counts from scEC&T-seq over the reconstructed MYCN ecDNA region in patient #2 single nuclei (h; two-sided Pearson correlation, P = 3.2e-13, R = 0.69, n = 86 patient #2 nuclei) and in patient #1 single nuclei (i; two-sided Pearson correlation, P = 7.6e-13, R = 0.66, n = 93 patient #1 nuclei).

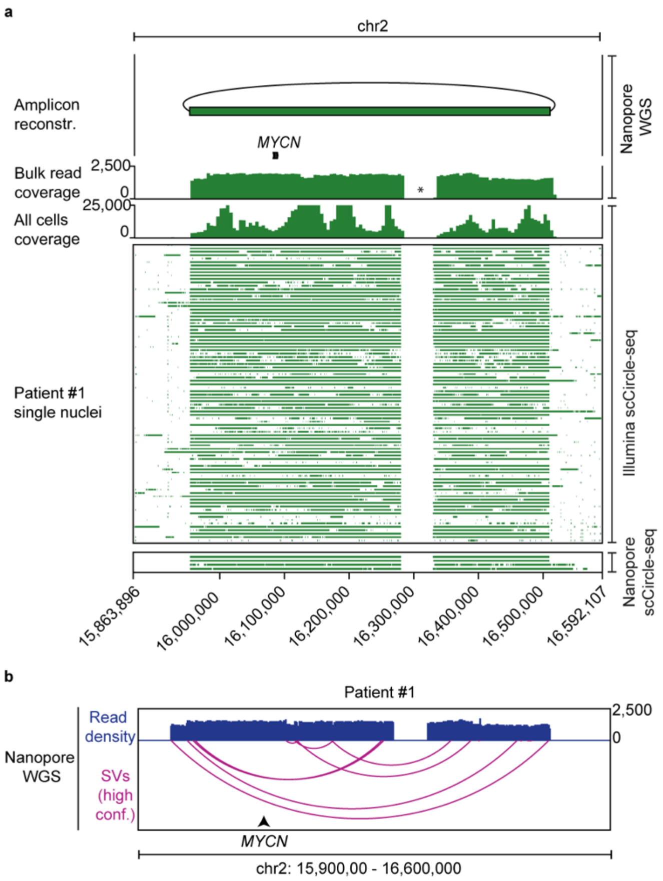

Supplementary Figure 17. Patient #1 presents a complex ecDNA structure with multiple internal rearrangements. a, Simplified long-read based amplicon reconstruction derived from WGS sequencing data in bulk cell populations and read coverage over the ecDNA region across single cells in patient #1 (*n* = 93 nuclei) as detected by scEC&T-seq. Top to bottom: simplified ecDNA amplicon reconstruction, copy number profile, gene annotations, read density over the ecDNA region in merged single cells, coverage over the ecDNA region in single cells (rows) as detected by short or long-read scEC&T-seq. **b,** Genome track of long-read nanopore WGS data displaying read density across the *MYCN* ecDNA region in patient #1, and showing the identified high-confidence SVs within the ecDNA amplicon region.

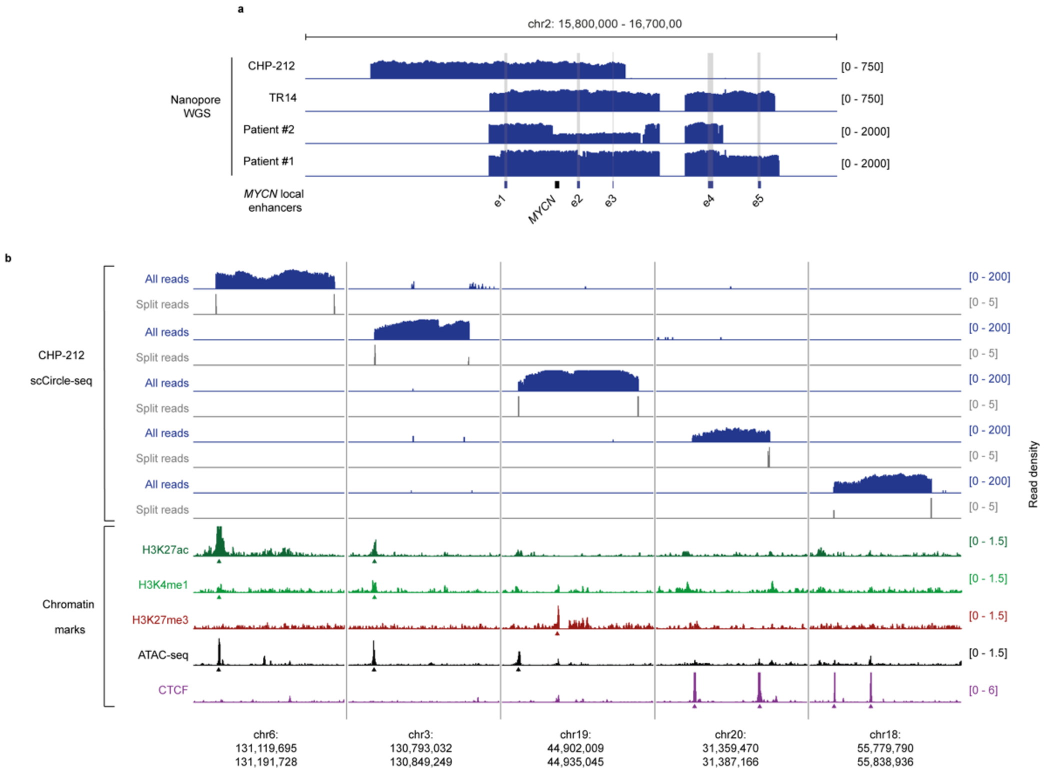

Supplementary Figure 18. ***MYCN*** **local enhancers are recurrently included in ecDNA in neuroblastoma. a,** Genome tracks of long-read nanopore WGS data showing co-amplification in ecDNA of *MYCN* local enhancers (e1-e5) with the *MYCN* proto-oncogene in neuroblastoma cell lines (CHP-212 and TR14) and primary tumors (patient #1 and #2). **b,** Overlap of non-recurrent extrachromosomal circular DNAs with chromatin marks. Top to bottom: 5 exemplary genome tracks of 5 different CHP-212 single cells (Log-scaled total read density in blue and circle edge read density in grey); H3K27ac ChIP-seq (dark green); H3K4me1 ChIP-seq (light green); H3K27me3 ChIP-seq (red); ATAC-seq (black); CTCF ChIP-seq (purple).

Supplementary Table 7: Oligos used in scEC&T-seq protocol

**Primer Name**	**Primer Sequence**	**Provider**
Oligo-dT	5′-biotin-triethyleneglycol-AAGCAGTGGTATCAACGCAGAGTACT30VN-3′,	IDT
Template Switch Oligo (TSO)	5′-AAGCAGTGGTATCAACGCAGAGTACrGrG+G-3′, where "r" indicates a ribonucleic acid base and "+" indicates a locked nucleic acid base	IDT
ISPCR	5′-AAGCAGTGGTATCAACGCAGAGT-3	IDT

Supplementary Note 1: QC results from scEC&T-seq data in cell lines, tumor nuclei and T-cells

Based on the observation that longer exonuclease exposure improved small circular DNA enrichment and recovered large ecDNA, we used sequencing data from cells digested with exonuclease for 5 days for downstream analyses in all cases. In cell lines, a total of 182 cells (154 CHP-212 cells and 28 TR14 cells) were exposed to exonuclease digestion for 5 days. Of those, 175 cells (96.15%; 150 CHP-212 and 25 TR14 cells, Supplementary Table 1) passed scCircle-seq quality control. Of those cells, 149 cells additionally passed scRNA-seq quality control (81.87%; 129 CHP-212 cells and 20 TR14, Supplementary Table 1 and 2). We detected on average 9,058 +- 1,163 full mRNA transcripts from different genes per cell (9,328 +- 1,006 in CHP-212 and 7,961 +- 1,124 in TR14; Supplementary Fig. 3b and Supplementary Table 2). For a total of 94 single T-cells (47 per patient) and 190 primary tumor nuclei (95 per primary tumor sample), the DNA was exposed to exonuclease digestion for 5 days in all cases. Of those, 79 T-cells (84%; 38 patient #3 and 41 patient #4) and 179 nuclei (93 patient #1 and 86 patient #2) passed quality control criteria (Supplementary Table 1 and 2). We identified 3,793 +- 1,055 mRNA transcripts per cancer cell nucleus (Supplementary Fig. 15c; Supplementary Table 2) and 3,177 +- 541 transcripts per T-cell (Supplementary Fig. 15d; Supplementary Table 2). Both CD4+ and CD8+ T-cells were identified based on their expression profiles (Supplementary Fig. 15l-n). Unsupervised clustering of tumor nuclei separated two population based on patient origin (Supplementary Fig. 15o). In both cancer nuclei populations, we observed expression of common neuroblastoma cell markers, including *MYCN, PHOX2B, HAND2, ALK* and *GATA3* (Supplementary Fig. 15p-t).
