# Supplementary material for: Parallel sequencing of extrachromosomal circular DNAs and transcriptomes in single cancer cells
Source: Nat Genet. 2023 May 4;55(5):880–90. doi: 10.1038/s41588-023-01386-y (PMC10181933; doi:10.1038/s41588-023-01386-y)
Supplement: Supplementary file 2 — Reporting Summary [file 41588_2023_1386_MOESM2_ESM.pdf]

nature portfolio

Corresponding author(s): Anton G. Henssen

Last updated by author(s): 23/02/23

Reporting Summary

Nature Portfolio wishes to improve the reproducibility of the work that we publish. This form provides structure for consistency and transparency in reporting. For further information on Nature Portfolio policies, see our Editorial Policies and the Editorial Policy Checklist.

Statistics

For all statistical analyses, confirm that the following items are present in the figure legend, table legend, main text, or Methods section.

n/a	Confirmed	
☐	☒	The exact sample size (*n*) for each experimental group/condition, given as a discrete number and unit of measurement
☒	☐	A statement on whether measurements were taken from distinct samples or whether the same sample was measured repeatedly
☐	☒	The statistical test(s) used AND whether they are one- or two-sided<br>*Only common tests should be described solely by name; describe more complex techniques in the Methods section.*
☒	☐	A description of all covariates tested
☒	☐	A description of any assumptions or corrections, such as tests of normality and adjustment for multiple comparisons
☐	☒	A full description of the statistical parameters including central tendency (e.g. means) or other basic estimates (e.g. regression coefficient) AND variation (e.g. standard deviation) or associated estimates of uncertainty (e.g. confidence intervals)
☐	☒	For null hypothesis testing, the test statistic (e.g. *F*, *t*, *r*) with confidence intervals, effect sizes, degrees of freedom and *P* value noted<br>*Give P values as exact values whenever suitable.*
☒	☐	For Bayesian analysis, information on the choice of priors and Markov chain Monte Carlo settings
☒	☐	For hierarchical and complex designs, identification of the appropriate level for tests and full reporting of outcomes
☐	☒	Estimates of effect sizes (e.g. Cohen's *d*, Pearson's *r*), indicating how they were calculated

Our web collection on statistics for biologists contains articles on many of the points above.

Software and code

Policy information about availability of computer code

Data collection	FACSAria Fusion flow cytometer (Biosciences)
Data analysis	FIJI (version 2.1.0), Trim Galore (version 0.6.4), Bwa mem (version 0.7.17 and version 0.7.15), Hisat2 (version 2.2.1), rsem (version 1.3.1), Guppy (version 5.0.14), NanoFilt (version 2.8.0), ngmlr (version 0.2.7), Sniffles(version 1.0.12) , deepTools (version 3.5.1) , FASTQC (version 0.11.8), Picard (version 2.20.4 and 2.16.0 and 2.23.8), BBMap (version 38.58), regioneR(version 1.24.0), Homer (version 4.11), Lumpy-sv(version 0.2.14 and 0.3.1), SvABA (version 1.1.0), Samtools (version 1.11 and 1.10), GATK4/Mutect2(version 4.1.9.0), Mutserve(version 2.0.0-rc12), AlleleCounter (version 4.0.2), ComplexHeatmap(version 2.6.2), dendextend (version 1.15.2), Seurat (4.10), clusterProfiler (4.0.5), STAR (version 2.7.9a), Arriba (version2.1.0), gGnome (version 0.1), gTrack(0.1.0), Genomic Ranges (1.44.0), ggplot (version 3.4.1) . Data analysis code associated with this publication can be found here: https://github.com/henssen-lab/scEC-T-seq

For manuscripts utilizing custom algorithms or software that are central to the research but not yet described in published literature, software must be made available to editors and reviewers. We strongly encourage code deposition in a community repository (e.g. GitHub). See the Nature Portfolio guidelines for submitting code & software for further information.

Data

Policy information about availability of data

All manuscripts must include a data availability statement. This statement should provide the following information, where applicable:

- Accession codes, unique identifiers, or web links for publicly available datasets
- A description of any restrictions on data availability
- For clinical datasets or third party data, please ensure that the statement adheres to our policy

Sequencing data generated in this study are available at the European Genome-phenome Archive (EGA) under the accesion number: EGAS00001007026. ChIP-seq

narrowpeak and bigwig files from Helmsauer et al, were downloaded at https://data.cyverse.org/dav-anon/iplant/home/konstantin/helmsaueretal/. Additional source data files are provided with this paper. All other data are available from the corresponding authors upon reasonable request.

Field-specific reporting

Please select the one below that is the best fit for your research. If you are not sure, read the appropriate sections before making your selection.

☒ Life sciences ☐ Behavioural & social sciences ☐ Ecological, evolutionary & environmental sciences

For a reference copy of the document with all sections, see nature.com/documents/nr-reporting-summary-flat.pdf

Life sciences study design

All studies must disclose on these points even when the disclosure is negative.

Sample size	No statistical methods were used to predetermine sample size. Sample size was decided based on multiple factors including:<br>(1) The cell numbers used in previous publications from similar low-throughput single cell genomic methods, which allowed them to detect statistically significant differences between conditions.<br>(2) Cost<br>(3) Tissue samples availability
Data exclusions	No data was excluded for analyses
Replication	Biological replicates across two different cell lines and tissue samples showed consistent and reproducible experimental results. To exclude bacht effects and ensure reproducibility, we included and processed in parallel multiple experimental conditions and, when possible multiple cell lines, in different 96-well plates. FISH experiments were done once per cell line and primary tumor.
Randomization	There was no subject randomization in this study.
Blinding	Patients were deidentified and assigned a study-specific identification number. Investigators were not blinded to allocation during experiments and outcome assessment.

Reporting for specific materials, systems and methods

We require information from authors about some types of materials, experimental systems and methods used in many studies. Here, indicate whether each material, system or method listed is relevant to your study. If you are not sure if a list item applies to your research, read the appropriate section before selecting a response.

Materials & experimental systems

n/a	Involved in the study	
☐	☒	Antibodies
☐	☒	Eukaryotic cell lines
☒	☐	Palaeontology and archaeology
☒	☐	Animals and other organisms
☐	☒	Human research participants
☒	☐	Clinical data
☒	☐	Dual use research of concern

Methods

n/a	Involved in the study	
☐	☒	ChIP-seq
☐	☒	Flow cytometry
☒	☐	MRI-based neuroimaging

Antibodies

Antibodies used	Anti-H3K27me3 polyclonal antibody (Sigma-Aldrich, cat. # 07-449, lot #2382150 , dilution 1:480)
Validation	Anti-H3K27me3 (Sigma-Aldrich, cat. # 07-449, lot #2382150) has been validated for ChIP-seq by the manufacturer (https://www.abcam.com/histone-h3-tri-methyl-k27-antibody-epr18607-chip-grade-ab192985.html)

Eukaryotic cell lines

Policy information about cell lines

Cell line source(s)	Human tumor cell lines were obtained from the American Type Culture Collection (CHP-212; ATCC; Manassas, VA, USA) or were kindly provided by J. J. Molenaar (TR14; Princess Máxima Center for Pediatric Oncology, Utrecht, Netherlands).
Authentication	The identity of all cell lines was verified by STR genotyping (Genetica DNA Laboratories and IDEXX BioResearch)

Mycoplasma contamination	Absence of Mycoplasma sp. contamination was determined with a Lonza MycoAlert system (Lonza)
Commonly misidentified lines (See ICLAC register)	The cell lines included in this study (CHP-212 and TR14) are confirmed neuroblastoma cell lines. No commonly misidentified cell lines were used in this study.

Human research participants

Policy information about studies involving human research participants

Population characteristics	This study comprised the analyses of tumor and blood samples of patients diagnosed with neuroblastoma between 1991 and 2022. Age or gender is irrelevant to this study.
Recruitment	Patients were registered and treated according to the trial protocols of the German Society of Pediatric Oncology and Hematology (GPOH). This study was conducted in accordance with the World Medical Association Declaration of Helsinki (2013) and good clinical practice; informed consent was obtained from all patients or their guardians. No bias in recrutment is relevant to this study.
Ethics oversight	The collection and use of patient specimens was approved by the institutional review boards of Charité-Universitätsmedizin Berlin and the Medical Faculty, University of Cologne.

Note that full information on the approval of the study protocol must also be provided in the manuscript.

ChIP-seq

Data deposition

☒ Confirm that both raw and final processed data have been deposited in a public database such as GEO.

☒ Confirm that you have deposited or provided access to graph files (e.g. BED files) for the called peaks.

Data access links *May remain private before publication.*	ATAC-seq and H3K4me1, H3K27ac ChIP-seq narrowpeak and bigwig files from Helmsauer et al. were downloaded at https://data.cyverse.org/dav-anon/iplant/home/konstantin/helmsaueretal/. Sequencing raw data from Helmsauer et al are available at the Sequence Read Archive under accession PRJNA622577.<br><br>Generated H3K27me3 ChIP-seq raw data, as well as big wig and narrow peak bed files, are available at the European Genome-phenome Archive (EGA) under the accesion number: EGAS00001007026.
Files in database submission	The following data files are available for ATAC-seq, H3K4me1, H3K27ac and H3K27me3 under the data access links provided above:<br>- fastaq files<br>- bw files (hg19)<br>- narrow peaks bed files
Genome browser session (e.g. UCSC)	No longer applicable

Methodology

Replicates	This study is mainly based on published ChIP-seq data which had not been acquired in replicates.
Sequencing depth	ChIP-seq: 25M 75bp single-end reads
Antibodies	Anti-H3K27me3 polyclonal antibody
Peak calling parameters	MACS2 (2.1.2) with default parameters
Data quality	Data was quality controlled using RPC, NPC and composite plots over housekeeping genes.
Software	FASTQC 0.11.8, BBMap 38.58, BWA-MEM 0.7.15, Picard 2.20.4, Deeptools 3.3.0, MACS2 2.1.2

Flow Cytometry

Plots

Confirm that:

☒ The axis labels state the marker and fluorochrome used (e.g. CD4-FITC).

☒ The axis scales are clearly visible. Include numbers along axes only for bottom left plot of group (a 'group' is an analysis of identical markers).

☒ All plots are contour plots with outliers or pseudocolor plots.

☒ A numerical value for number of cells or percentage (with statistics) is provided.

Methodology

Sample preparation	CHP-212 or TR14 cells in culture were trypsinized, washed once with 1xPBS and resuspended in 1xPBS and Propidium Iodide (PI) for sorting. Peripherial blood mononuclear cells (PBMCs) were isolated using density gradient centrifugation with Ficoll resuspended and Propidium Iodide PI in 1xPBS for sorting. . PBMCs suspensions were additionally stained with a 1:400 dilution of anti-human CD3. Nuclei suspensions were stained with DAPI
Instrument	FACSAria Fusion flow cytometer (Biosciences)
Software	FlowJo 10.7.1
Cell population abundance	Cell percentages are shown in Supplementary figures 1a,b and 14a,b. CD3+ DAP- live, T-cell population abundance was 6% in patient #3 derived PBMCs and 14.5% in patient #4 derived PBMCs.
Gating strategy	In all cases, events were separated from debris using Forward scatter (FSC) vs. side scatter (SSC). To separate live from dead cells Forward scatter (FSC) vs. propidium iodide (PI) was used in cell lines (TR14 and CHP-212). CD3+ and DAPI- PBMCs were sorted. In the case of nuclei, DAPI+ nuclei were sorted.

☒ Tick this box to confirm that a figure exemplifying the gating strategy is provided in the Supplementary Information.
